# Clinical supervision in podiatry in Australia and New Zealand: supervisor challenges in this role

**DOI:** 10.1186/s12909-023-04056-z

**Published:** 2023-02-09

**Authors:** Katrina Reynolds, Michelle McLean

**Affiliations:** Faculty of Health Sciences and Medicine, Bond University, Level 2, Building 5, 14 University Drive, Robina, QLD 4226 Australia

**Keywords:** Clinical supervision, Training, Podiatry, Partnership

## Abstract

**Background:**

Clinical supervisors play an integral role in preparing podiatry graduates for clinical practice. Not enough is, however, known about how prepared podiatry clinical supervisors are for this role, in terms of training received and the challenges they face in the role. Informed by previous qualitative research, this study extends our understanding of what it means to be a clinical supervisor in podiatry.

**Methods:**

An online survey comprising closed and open-ended questions gathered data from 67 registered podiatrists who were also clinical supervisors. Descriptive analysis was undertaken. Chi-square analysis was used to test independence between preparedness for supervision and variables of interest (e.g. training received). Item analysis was assessed using Cronbach’s alpha coefficients and Kendall’s Tau to determine whether statistically significant associations existed across the broad challenges previously identified (i.e. supervisor-specific, curriculum and students). Open-ended comments were analysed using content analysis.

**Results:**

Generally, most supervisors (64%, 43/67) initially felt “ prepared” to supervise, despite the majority (58%, 39/67) not having received any training or educational support. Overwhelming, supervisors (97%, 65/67) considered universities responsible for ensuring quality clinical supervision. They perceived many of the previously identified supervisor-specific challenges (e.g. time-consuming), curriculum issues (e.g. limited hands-on patient contact in private practice placements) and student deficiencies (e.g. poor time management). Positive correlations were found across the three sets of challenges, with the strongest measure of association found between overall student deficiencies and overall curriculum issues (*p* < .001).

**Conclusion:**

These findings contribute to a deeper understanding of clinical supervision in podiatry. The study identified inconsistent support for clinical supervisors from partner universities. This study found a clear desire and need for supervisor training. A partnership approach is recommended in which universities work with clinical supervisors to address their overall challenges in terms of supervisor professional development, paying attention to curriculum issues, and improving student preparedness during placements.

**Supplementary Information:**

The online version contains supplementary material available at 10.1186/s12909-023-04056-z.

## Background

Clinical supervisors of pre-registration health professional students are not only responsible for assessing clinical competence in terms of patient safety and the quality of the patient experience [1, 2], but they also contribute to students’ professional development [3] including their professional identity [4]. For many registered podiatrists in Australia and New Zealand, taking on the role of clinical supervisor of undergraduate podiatry students is voluntary, often to meet continuing professional development requirements pertaining to community service [5]. In addition, the public health system in particular expects employees (at various levels) to provide clinical supervision to junior health professionals including undergraduate students from the same discipline. It is therefore likely that many registered and practicing podiatrists are appointed as clinical supervisors, with the assumption that as practitioners in their discipline [6], they will also supervise podiatry students on placements.

Being a clinical supervisor, however, necessitates an expansion of professional identity from clinician to the dual roles of clinician and supervisor [7]. For Watkins and Milne [4], clinical supervision should be recognised as a distinct, area of professional practice, with its own process, models and approaches. In their opinion, clinical supervisory skills do not “fall from the sky” (p.178). Training and professional development are required. Whilst training to be an effective clinical supervisor is considered essential amongst the health professions [8], approaches to supervisory training in podiatry have not been extensively documented. Informed by an earlier exploratory qualitative study (October 2015-May 2016) of clinical supervisors’ preparedness to supervise podiatry students in Australia [9], a larger sample of clinical supervisors was surveyed to further explore their experiences, preparedness, and training for the role, as well as previously identified curriculum issues and student preparedness for clinical placements. A greater understanding of these considerations will hopefully inform effective training and support for clinical supervisors, particularly considering that currently podiatry graduates in Australia and New Zealand (and in some countries internationally, e.g. United Kingdom, South Africa) enter clinical practice without an internship or clinical supervision. As podiatry graduates need to ‘hit the ground running’ as safe and competent health care providers, quality supervision during clinical placements should be an imperative.

The following research questions informed this study:

1. What training or educational support did clinical supervisors receive to prepare them for this role?
2. At supervision commencement, how prepared were clinical supervisors to supervise podiatry students?
3. In terms of previously identified challenges (self, curriculum, and student-related), which were most pressing for clinical supervisors?

## Methods

### Survey Design

A questionnaire was developed in Survey Monkey based on the first phase of this research, a qualitative study of 11 clinical supervisors [9]. Appendix 1 provides the survey instrument comprising demographic data (e.g. gender, age group, year of graduation as a podiatrist) plus three (3) sections:

1. Clinical supervision status, and/or interest in becoming a clinical supervisor.
2. Experiences as a clinical supervisor, including for example, years as supervisor; location of supervision (i.e. public, private sector); perceived level of preparedness to supervise podiatry students at commencement as a supervisor (Likert scale: 1 = Not at all prepared, 4 = Very prepared); receipt of any supervisory training or educational support (yes/no); and onus for ensuring quality clinical supervision (Likert scale: 1 = Not at all responsible, 4 = Completely responsible).
3. Supervisory challenges comprised 29 items (Likert scale: 1 = Strongly disagree, 5 = Strongly agree), measuring participant perception of previously identified sets of challenges: Supervisor-specific (*n* = 10) e.g. *supervision of students is time-consuming*; curriculum issues (*n* = 8) e.g*. placement is too short*; and, student deficiencies (*n* = 11) e.g. *students lack confidence in their clinical skills.*

Questionnaire format, sequence, wording and length were carefully considered to ensure reliability, validity and sustained participant engagement [10, 11]. A combination of closed (including Likert scale items) and open-ended questions (comment boxes) were included. The survey was piloted (i.e. for face and content validity) with two registered podiatrists who were clinical supervisors (excluded from the data analysis). Pilot participants provided feedback on content and format, including language suitability in item wording and intention, to allow minor survey refinement for clarity [12].

### Sample and administration

Through purposive and snowball sampling, registered and practising podiatrists were invited to voluntarily participate in this second phase larger research study, which was designed to allow a sub-set of participants, the clinical supervisors to answer additional questions (one at a time) relating to student supervision. Clinical supervisors were identified as either current or past clinical supervisors of podiatry students in public or private sector and/or at Australian and New Zealand universities. Approval to conduct the study was obtained from the Bond University Human Research Ethics Committee (RO15226). Initially, advertisements were placed on the Australian Podiatry Association and PodiatryNZ social media member platforms. A search for podiatrists and their contact details in general practice databases was also undertaken. Potential participants were invited by email which provided the survey link, a cover letter outlining ethical approval and describing the study as a two-part investigation, i.e. Their preparedness for podiatry practice (article in preparation) and student supervision (current study). The time commitment for completing the survey being approximately 20 min. Data were collected between December 2018 and May 2019. Two reminder emails were sent. Submission of the survey implied a respondent’s consent to participate in the study. Although 80 respondents completed the first item of the survey, 13 were not clinical supervisors of podiatry students on placement. Of these, 46% had no interest in becoming a supervisor. Sixty-seven respondents were included in data analyses as having completed the survey in its entirety (83.8% response rate).

### Data analyses

Descriptive statistics were used to analyse the responses to each survey item. Chi-square tests were used to examine differences in proportions and associations between preparedness to supervise, and, selected variables of interest, such as gender, years practising as a podiatrist, years supervising, expectations to supervise, and training received. To enable subsequent application of statistical test methods like chi-square test and for ease of interpretation purposes, some questions were collapsed into dichotomous variables, e.g. age groups (i.e. ‘44 and under’, ‘45 and above’), ‘prepared to supervise’ – “somewhat prepared” or better (i.e. “very prepared”, *vs.* ‘unprepared to supervise’ – “somewhat unprepared” or worse (i.e. “not at all prepared”); years practising as a podiatrist – “pre-1999” *vs.* “post 2000”; years supervising – “5 years or less” *vs.* “5 years or more”. Cronbach’s alpha was used to measure the internal consistency of the items within each challenge category, with a reliability coefficient of 0.60 or greater considered acceptable [13]. To test whether there was any significant relationship (and strength of correlation) between two independent items relating to challenges, Kendall’s tau were used given the small data set [14]. Since these were ordinal variable (i.e. Likert scale), this method was considered appropriate [15]. As comparisons of individual challenges in one or more tables would be very cumbersome, we chose to compute a mean variable from each challenge set to create overall variable mean scores, and the same with the total items variable score (i.e. 29 items, overall mean) for a more concise interpretation of these data. All statistical analyses were performed using SPSS V.28.0. Statistical significance was defined as *p* < 0.05.

Free text comments were analysed using content analysis [16] to enable simple reporting of common issues and given the exploratory nature of our research [17]. Initial identification of coding categories was undertaken by the principal researcher (KR) becoming familiar by reading and re-reading the qualitative data. An iterative process of categorising and re-categorising was involved. Manageable code categories were then sorted into themes. Both researchers (KR & MM) regularly discussed the analysis to ensure consistency and agreement through the process and to permit finalising for reporting.

### Results

#### Demographic profile of respondents

Table 1 summarises the demographic profile of respondents. For the 67 respondents, 62.7% were female, 58.2% were aged between 35 and 54, while 50.7% had completed a Bachelor qualification in podiatry. Seventy-six percent had qualified in Australia, with the median (range) year of graduation as a registered podiatrist being 1999 (1969–2017). Mean years practicing as a registered podiatrist was 20.8 (*SD* = 11.93), with the most recent to become a clinical supervisor having two years of registered practice and one to six months of clinical supervision. Of those with additional qualifications, around 29% had undertaken education-related studies (e.g. training and assessment certificate, diabetes education, tertiary education). For approximately two-thirds (65.7%), clinical supervision was expected in their workplace. Many individual respondents provided clinical supervision in both the public and private sectors, supervising students in different year levels (Years 1–4) in either a Bachelor or a graduate-entry Masters degree. The majority (62.7%) had been supervising for more than 5 years. While 67.2% were planning to continue supervising, 19.4% were unsure, mainly due to a lack of support from the partnering university, too little time, and/or perceived poor student attitude.

**Table 1:** Participant demographics (*n* = 67)

Characteristics	Total responses *n* (%)
**Gender**	
Male	25 (37.3)
Female	42 (62.7)
**Age group**	
18–24	1 (1.5)
25–34	13 (19.4)
35–44	18 (26.9)
45–54	21 (31.3)
55–64	10 (14.9)
65–74	4 (6.0)
**Initial podiatry qualification**	
Associate Diploma	7 (10.4)
Diploma	19 (28.4)
Bachelor	34 (50.7)
Bachelor with Honours	5 (7.5)
Master (Graduate-entry)	2 (3.0)
**Location of qualification**	
Australia	51 (76.1)
New Zealand	10 (14.9)
Other^a^	6 (9.0)
**Other formal qualifications**	
Yes^b^	35 (52.2)
No	32 (47.8)
**Years of supervision experience**	
< 1 month	2 (3.0)
1–6 months	6 (9.0)
1–2 years	7 (10.4)
2–5 years	10 (14.9)
> 5 years	42 (62.7)
**Last time supervision provided**	
Past week	8 (11.9)
Past month	4 (6.0)
2–3 months	18 (26.9)
4–6 months	10 (14.9)
7–12 months	7 (29.9)
1 or more years	20 (29.9)
**Better environment to supervise – public or private sector**	
Public	18 (26.9)
Private	2 (3.0)
There is no difference	9 (13.4)
Unsure	5 (7.5)
Not applicable^c^	33 (49.3)
**Work setting for supervision**^d^	**86 (100.0)**
Public sector hospital	32 (37.2)
Private sector hospital	1 (1.2)
Private practice	28 (32.6)
Community health centre (public sector)	14 (16.3)
University student clinic (purpose-built)	11 (12.8)
**Student year supervised**^d^	**115 (100.0)**
1^st^ year	6 (5.2)
2^nd^ year	16 (13.9)
3^rd^ year	45 (39.1)
4^th^ year	45 (39.1)
Unsure	3 (2.6)
**Qualification in which students enrolled**^d^	**96 (100.0)**
Bachelor	60 (62.5)
Honours	12 (12.5)
Graduate-entry (Bachelors)	8 (8.3)
Graduate-entry Masters	8 (8.3)
Masters	5 (5.2)
Doctoral (Doctor or Podiatric Medicine)	1 (1.0)
Unsure	2 (2.1)

^a^ Other (United Kingdom *n* = 4, South Africa *n* = 2)

^b^ Other formal qualifications (varied e.g. nursing, wound management etc.)

^c^ Did not provide supervision in both to provide comparison

^d^ Based on multiple answer question

#### Training and educational support received

In terms of training or educational support, 58.2% (39/67) had received none from the partnering institution. For many, the only exchange was the university’s initial request to supervise:

*“I received a request to supervise students for clinical placement and was happy to do this. No other support or information has ever been forthcoming, except receiving names and dates of students who had been assigned to me”* [Respondent 38F]

For those who indicated that they had received training, for the most part this ‘training’ was minimal, ranging from material provision (e.g. initial information, assessment forms) to sporadic one-day sessions:

*“The University provided a one-day session several years ago”* [Respondent 66F]

Because training was minimal or non-existent, a number independently completed training and/or an education-based qualification to better support them in their supervisory role:

*“After supervising for some time, I was enrolled in a course to support my supervision, however, there is limited education available to clinicians even now”* [Respondent 43F]

**Fig. 1:**
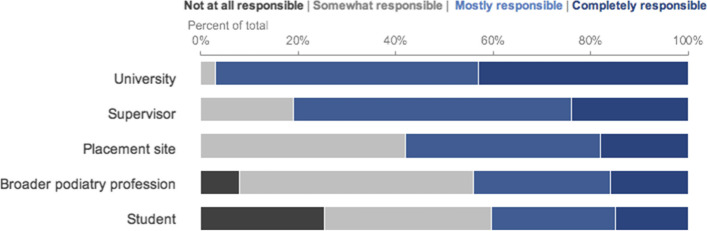
Distribution (%) of responses to question on whose responsibility it is to ensure quality clinical supervision

Clinical supervisors were unanimous (97%) that the university was primarily responsible for ensuring quality clinical supervision, followed by the individual supervisor (80.6%). Internal consistency (of the 5 items) was 0.70, indicating reliability (Fig. 1).

#### Initial preparedness/unpreparedness to supervise

In response to *“When you commenced supervision, how prepared were you to supervise podiatry students?”,* 35.8% were unprepared (13.4% not at all prepared; 22.4% somewhat unprepared), while 64.2% were generally prepared (41.8% somewhat prepared; 22.4% very prepared). No statistically significant associations (Chi-square test of independence) were found when initial preparedness to supervise (i.e. dichotomous variable) was compared with gender, age group, other formal qualifications, years practicing, years supervising, expectations to supervise, and with training received to supervise.

Based on the 21 respondent comments for this item (Q29), three themes (with some overlap) were identified in terms of their related ratings of preparedness to supervise podiatry students which included: *Supervisory training at the outset*, *professional clinical experience*, and *prior experiences as a student*. In terms of *supervisory training at the outset*, many described having been involved in various forms of relevant training:

*“I had TAFE Cert IV [Certificate IV in Training and Assessment] qualifications and have taught various other programs to health professionals most of my working life”* [Respondent 22F – very prepared]

For many, their *professional clinical experience* contributed to them feeling “prepared” despite no preparatory training prior to commencing:

*“There was no formal training or support – I was prepared from my own clinical work prior to the student commencing”* [Respondent 25F – somewhat prepared]

One respondent, however, showed no apparent interest in training, relying solely on professional clinical experience:

*“Had been in private practice for many years…There was a course available at TAFE [Technical and Further Education], I did not do it”* [Respondent 13M – very prepared]

Others, including the most recent clinical supervisor of two months, used their *own prior experiences* of being supervised as students as preparation to supervise. Common to these was no other mention of formal supervisory training engagement:

*“Due to being a few years out myself, I recall what it is like to be a student and the qualities of my clinical placement that were important for my learning. I mirrored my clinical supervisors’ teaching methods…However, I am unsure if it is suitable [being a supervisor] due to myself only working for two years”* [Respondent 8F – somewhat prepared]

The remaining comments (*n* = 6) provided explanation for respondents otherwise feeling ‘unprepared’ initially to supervise, and much of this was due to either not having received training or support, having had no initial choice in the matter (e.g. *“…just thrust upon you*” [R21M]), and/or not feeling comfortable with their own clinical knowledge to in fact supervise.

#### Supervisory challenges, curriculum issues and student deficiencies

Table 2 shows respondents’ level of agreement for three broad sets of challenges. Cronbach’s alpha reliability coefficient for each set of challenges was *supervisor-specific* (α = 0.76), *curriculum issues* (α = 0.62), and *student deficiencies* (α = 0.86). The overall internal consistency (α = 0.86) confirmed high reliability. An overall perception score, ranging from 1 to 5, was calculated using the mean (SD) of all challenge item ratings for each respondent.

**Table 2:** Ratings for 29 items for three previously identified groups of challenges (Reynolds & McLean, 2022) (*n* = 67)

		***n* (%)**					
	**Challenges identified**	**SD**	**D**	**N**	**A**	**SA**	**Total agree**
***Item***	***Supervisor-specific (n* = *10)***						
33a	Supervision of students is time-consuming	3 (4.5)	0 (0.0)	7 (10.4)	34 (50.7)	23 (34.3)	57 (85.0)
35e	There is limited university support and/or resource for supervisory role	1 (1.5)	5 (7.5)	20 (29.9)	26 (38.8)	15 (22.4)	41 (61.2)
35b	Lack of standardised assessment criteria between universities creates difficulties for supervisors	1 (1.5)	5 (7.5)	20 (29.9)	26 (38.8)	15 (22.4)	41 (61.2)
35c	Lack of formal and/or informal training contributes to uncertainty as a supervisor	2 (3.0)	7 (10.4)	18 (26.9)	25 (37.3)	15 (22.4)	40 (59.7)
33c	Supervisors experience fatigue, stress and burnout from students	2 (3.0)	9 (13.4)	19 (28.4)	29 (43.3)	8 (11.9)	37 (55.2)
35a	Universities do not provide clarity of the supervisory role, related responsibilities and processes	3 (4.5)	9 (13.4)	21 (31.3)	23 (34.3)	11 (16.4)	34 (50.7)
35d	There is limited workplace support and/or resource for supervisory role	1 (1.5)	10 (14.9)	25 (37.3)	18 (26.9)	13 (19.4)	31 (46.3)
33b	When providing patient care, it is difficult to balance being a good clinician and an effective supervisor	8 (11.9)	16 (23.9)	14 (20.9)	23 (34.3)	6 (9.0)	29 (43.3)
33d	Patients become fatigued from students	4 (6.0)	18 (26.9)	23 (34.3)	17 (25.4)	5 (7.5)	22 (32.9)
33e	It is difficult to keep up-to-date with own knowledge and skills in clinical practice to be an effective supervisor	9 (13.4)	29 (43.3)	18 (26.9)	11 (16.4)	0 (0.0)	11 (16.4)
	***Curriculum (n* = *8)***						
38d	There is a lack of training in small business administration skills for practice	1 (1.5)	2 (3.0)	19 (28.4)	25 (37.3)	20 (29.9)	45 (67.2)
38c	Students have limited contact with patients (despite the prescribed 1000 h of placement)	0 (0.0)	12 (17.9)	10 (14.9)	27 (40.3)	18 (26.9)	45 (67.2)
37d	Students have limited ‘hands-on’ patient contact in private practice placements which impacts student learning	1 (1.5)	2 (3.0)	19 (28.4)	21 (31.3)	24 (35.8)	45 (67.1)
38a	There is poor alignment of placement for the student’s stage of learning	0 (0.0)	5 (7.5)	26 (38.8)	24 (35.8)	12 (17.9)	36 (53.7)
38b	Clinical placement occurs too late in the curriculum	0 (0.0)	16 (23.9)	14 (20.9)	19 (28.4)	18 (26.9)	37 (55.3)
37e	Students are being exposed to more public sector “hands-on” placements which is causing an imbalance in learning	6 (9.0)	11 (16.4)	23 (34.3)	20 (29.9)	7 (10.4)	27 (40.3)
35f	Placement is too short (e.g. 1–2 weeks)	5 (7.5)	18 (26.9)	23 (34.3)	15 (22.4)	6 (9.0)	21 (31.4)
35 g	Placement is too long (e.g. 4–6 weeks)	4 (6.0)	24 (35.8)	30 (44.8)	8 (11.9)	1 (1.5)	9 (13.4)
	***Student deficiencies (n* = *11)***						
36c	Have poor time management skills	0 (0.0)	7 (10.4)	17 (25.4)	24 (35.8)	19 (28.4)	43 (64.2)
36d	Have limited ability to prioritise patient care	0 (0.0)	4 (6.0)	20 (29.9)	28 (41.8)	15 (22.4)	43 (64.2)
37c	Show limited ability to carry out basic manual podiatry clinical skill procedures	0 (0.0)	11 (16.4)	19 (28.4)	25 (37.3)	12 (17.9)	37 (55.2)
36a	Lack confidence in their clinical skills	1 (1.5)	9 (13.4)	21 (31.3)	27 (40.3)	9 (13.4)	36 (53.7)
36e	Lack independence when providing patient care	0 (0.0)	10 (14.9)	22 (32.8)	24 (35.8)	11 (16.4)	35 (52.2)
34c	Display a “know it all already” attitude on placement	3 (4.5)	20 (29.9)	17 (25.4)	17 (25.4)	10 (14.9)	27 (40.3)
34a	Lack interest and motivation on placement (e.g. marking time)	2 (3.0)	17 (25.4)	20 (20.9)	25 (37.3)	3 (4.5)	28 (41.8)
34b	Have unrealistic expectations about their intended learning during placement	2 (3.0)	19 (28.4)	19 (28.4)	22 (32.8)	5 (7.5)	27 (40.3)
37a	Are unable to communicate effectively with supervisors, other staff and patients	0 (0.0)	18 (26.9)	30 (44.8)	17 (25.4)	2 (3.0)	19 (28.4)
36b	Display difficulty ‘fitting in’ the clinical setting with staff and patients	1 (1.5)	23 (34.3)	25 (37.3)	13 (19.4)	5 (7.5)	18 (26.9)
37b	Have difficulty establishing rapport with patients	2 (3.0)	26 (38.8)	23 (34.3)	11 (16.4)	5 (7.5)	17 (23.9)

Likert 5-point scale, where 1 = strongly disagree (SD), 2 = disagree (D), 3 = neutral (N), 4 = agree (A), and 5 = strongly agree (SA)

In terms of *supervisor-specific challenges,* the time-consuming nature of supervision was the main challenge (85%), followed by the limited support they received (61.2%) and the lack of standardised assessment (61.2%). With respect to *curriculum issues*, the three most frequently identified challenges were: A lack of training in small business administration skills for practice (67.2%), students having limited contact with patients despite the prescribed 1000 h of placement (67.2%) and students having limited ‘hands-on’ patient contact in private practice placements, impacting on learning (67.1%). In terms of *student deficiencies*, the two main challenges were poor time management (64.2%) and their limited ability to prioritise patient care (64.2%).

#### Relationship across the three broad sets of challenges

Kendall’s Tau correlations across each of the three sets of challenges had a positive intercorrelation range between 0.18 to 0.49 (*p* =  < 0.05). The correlations were weak to strong, highlighting the unique contributions of each set of challenges in understanding supervisory challenges overall (Table 3). Weak, but significant, Kendall’s tau coefficients were found for *curriculum issues* and *supervisor-specific* (*n* = 67, τ_b_ = 0.182, *p* = 0.04); moderate, but significant for *student deficiencies* and *supervisor-specific* (*n* = 67, τ_b_ = 0.216, *p* = 0.01); and strong, and significant for *student deficiency areas* and *curriculum-related issues* (*n* = 67, τ_b_ = 0.486, *p* =  < 0.001). The total items score correlations with the three sets of challenges were either at the 0.50 level, with one exceeding the 0.60 level. All correlations were less than 0.001 level of probability, indicating that even the weakest of relationships was significant.

**Table 3:** Kendall rank correlation coefficients for comparing overall challenge items scores

**Themed items**		**Overall supervisory challenges**	**Overall curriculum issues**	**Overall student deficiencies**	**Total items score**
Overall supervisory challenges	r	1.000			
	t	-			
Overall curriculum issues	r	0.182^a^	1.000		
	t	0.039	-		
Overall student deficiencies	r	0.216^a^	0.486^a^	1.000	
	t	0.013	< 0.001	-	
Total items score	r	0.504^a^	0.590*	0.685*	1.000
	t	< 0.001	< 0.001	< 0.001	-

^a^Correlation is significant at the 0.05 level (2-tailed)

### Discussion

This study provides a snapshot of the challenges of being a podiatry clinical supervisor in Australia and New Zealand. Clinical supervisors in this follow-up study perceived many of the supervisor-specific (e.g. supervision of students is time consuming), curriculum issues (e.g. students have limited contact with patients) and student deficiencies (e.g. poor time management skills) as challenges that had been previously identified in the qualitative study [9].

### Supervisor-specific challenges

When presented with a range of supervisor-specific challenges, foremost was *the time-consuming nature* of clinical supervision, which is consistent with the literature across healthcare professionals. Rothwell et al. [18] in their evidence review, classified ‘lack of time’ as one of the main problems impacting quality clinical supervision. Competing clinical duties can also play a part in supervisors not being able to ‘find time’ for clinical supervision [19]. The second important challenge relates to a lack of adequate support and resources (i.e. *limited university support and/or resource for supervisory role*), which when coupled with the third challenge of a *lack of standardised assessment criteria between universities* likely places unnecessary pressure on the supervisor, possibly forcing them to ‘figure things out’ for themselves [18], rather than having clarity from specific guidance and/or guidelines to fulfil the role adequately [20]. Clinical supervisors, therefore require both pedagogical and organisational resources to be able to focus on patient care and student learning [21]. From these findings, it was no surprise that most respondents also agreed with the statement: *Supervisors’ experience fatigue, stress and burnout from students,* which support the Hyrkäs study of clinical supervision in psychiatric nurses, in which burnout (and low self-efficacy) was associated with a lack of support and supervisory dissatisfaction [22], and Rothwell and colleagues conclusion that a lack of ongoing support and engagement from leadership (e.g. the university) and organisation (e.g. the workplace) are real barriers to effective clinical supervision [18].

Surprisingly, while most respondents had received little or no training when they began supervising, they generally felt prepared for the role. For some, this related to further education and training they had already undertaken while others relied on their years of experience in clinical practice. For the more recent graduates (least supervisory experience), they drew on their personal experience as students being supervised. Whilst there was no statistical association between initial preparedness to supervise and years practicing, holding an opinion that years of podiatry practice is sufficient to supervise is concerning as it may hamper any pursuit and/or involvement in supervisor professional development, as these supervisors may not see the value of continuing professional development as a supervisor and could reflect a false impression of what is required to be an effective supervisor.

As Higgs and McAllister confirm, despite the role of ‘teaching’ being inherent in clinical practice (e.g. educating patients), when becoming a clinical supervisor, the clinician must transition into a new culture and set of responsibilities through organised training [23]. Further, relying on behavioural role modelling from a past supervisory relationship as means to supervise by emulating their actions [24], can be severely limited, particularly given individuals learn in different ways [25]. Whilst this respondent comment likely stemmed from an effective supervisor-supervisee relationship, as Sellers et al. [26] emphasise, problems in a past supervisory relationship for others may lead to, for example, an avoidance of becoming a supervisor due to a history of aversive interactions (and perhaps explanation for respondents indicating ‘no’ interest in becoming a supervisor in our initial sample). Without explicit training about learning and supervisory styles, the clinical supervisor would likely not appreciate the need to adjust this ‘preferred’ style in response to the perceived learning style of another student [25]. Such findings therefore raise opportunity for future research specific to the influence of supervisory styles and valued supervisory relationships on supervision outcomes in podiatry education. On a separate note, two years post registration to become a supervisor, begs additional enquiry around the appropriate number of years out practising prior to becoming a supervisor. In psychology, for example, an individual is not eligible to supervise unless they have held general registration for at least three years, and successfully completed full supervisory training with a board approved provider [27].

Indeed, many did identify that a lack of supervisory training and/or support resulted in their initial unpreparedness to supervise (despite also the years of clinical practise already behind them). This was found in the qualitative comments, confirming our earlier research, i.e. “Those who discussed receiving no supervision training or related professional development support also felt that they were not prepared for their supervisory roles” [9]. There was also general agreement with the statement that a *Lack of formal and/or informal training contributes to uncertainty as a supervisor*. As Martin et al. [2] explain, a lack of training in supervision is said to contribute to confusion regarding the supervision process, and undertaking training in supervision can assist supervisor preparedness in keeping up-to-date with evidence regarding effective clinical supervision practices. In fact, some clinical supervisors in the current study, mentioned later having undertaken formal training in supervision to assist their professional role development. Such actions reinforce how supervisory skills should not be taken for granted by virtue of experience, with training necessary to ensure that when clinicians become supervisors they also become skilled to supervise [2]. A more consistent approach to clinical supervisory training, preferably based on continuing appraisal of supervisors’ educational needs [28], is, however, necessary given participants’ minimal or varied training and/or support.

### Curriculum challenges

Supervisors perceived a lack of “*hands-on*” hours for students to practice their clinical skills in the private practice setting, and with patient contact generally, despite the prescribed 1000 h of placement. It is well-known that “*hands-on*” clinical exposure allows the application of theory to practice and promotes clinical skill development and self-efficacy [29]. *Lack of training in small business administration* for students was also a highly rated issue amongst respondents, a finding supported by McAllister and Nagarajan [30] of newly graduated allied health professionals not having much understanding of business practice. Bearing in mind that most podiatrists in Australia and New Zealand are in private practice (i.e. small business organisations), it would be particularly beneficial for final year students to have knowledge of the common administrative requirements necessary in practice prior to graduation.

### Student deficiencies

The main deficiencies identified were *poor time management* and* a limited ability to prioritise patient care*. Specifically, time management is about how one manages self [31], and for setting priorities, planning and implementing these in the available time [32]. Ghiasvand et al. [33] reinforce how time management skills are essential for clinical competence development, and Marthur et al. [32] assert time management can be successfully achieved by setting realistic goals, and by learning and consistent practice. Likewise, the agreement ratings associated with *students show limited ability to carry out basic manual podiatry clinical skill procedures, lack confidence in their clinical skills* and *lack independence when providing patient care,* indicate much need for improving podiatry students’ clinical skill experience and performance during clinical placements to ensure safe and independent practice at graduation. As Porter et al. [34] emphasise in nursing student education, self-confidence is a key component for effective clinical performance, and from more exposure to clinical placement, confidence and motivation levels will only improve. From our findings, it is clear that universities have a responsibility to produce graduates who are both competent and confident for practice in the clinical setting [34], and further research is thus recommended to identify causes of poor self-confidence in podiatry students on placement given the implications.

As the strongest statistical association (in terms of Kendall’s tau) is seen with *overall student deficiencies* and *overall curriculum issues*, the implication of this finding highlights the increased strain this is likely to place on clinical supervisors. Since the university needs to meet accreditation standards for podiatry training, it goes without saying that this commands quality supervision and as such requires the institution to ensure that all clinical supervisors meet minimum standards though providing the time, resources and training. Addressing the curriculum and student challenges should also be addressed via more focused hands-on clinical training at the very least.

### Limitations of the study

This study is limited by a relatively small sample size of podiatry clinical supervisors. The bulk of our sample was, however, representative of supervisors having been in this role for many years to garner an understanding of clinical supervisor experience in Australia and New Zealand. It is noted that since our data were collected, COVID-19 has had a profound impact presenting as a major challenge on its own for the profession [35]. With many placements cancelled at the onset of the pandemic and significant adjustments made, negatively impacting the clinical learning of students across the health professions [36]. Hence, whilst these findings were in agreement with and complement those from our previous research, a heightened awareness of specific supervisory challenges (even without the additional challenges of a pandemic) must be duly considered to inform future clinical supervisor preparedness, prior to and during supervisory practice.

## Conclusions

This study contributes to a deeper understanding of what it means to be a podiatry clinical supervisor pre-COVID. Training is clearly necessary to support the initial and ongoing role of the clinical supervisor (and to foster their professional identity development), particularly given clinical supervision cuts to the heart of professional podiatry training. Many of the challenges identified [9] were perceived by clinical supervisors, including the time-consuming nature of student supervision, along with role uncertainty likely stemming from limited university support and a lack of standardised assessment criteria creating difficulties for supervisors. Similarly, these clinical supervisors were in agreement with a number of previously identified curriculum issues: primarily students having limited hands-on contact with patients, which from this (and to be expected) causing student deficiency areas requiring further attention, specific to poor time management skills, inability to prioritise patient care, and a lack of independence and confidence in their clinical skills. These findings shed light on the complexity of the supervisor role. With the majority of clinical supervisors feeling initially *‘somewhat prepared’* to undertake their supervisory role, coupled with conditions that could limit good learning environments for the students they supervise, it is more than apparent that the universities must step up to support clinical supervisors, but, registered podiatrists who undertake the role of clinical supervisor must also share responsibility to upskill. The profession should consider minimum criteria for clinical supervisors, similar to the clinical competencies established for entry-level graduates, which in our opinion might include for example, accrual of a specific number of continuing education points for attendance at regular clinical supervision training sessions and/or encouragement for tertiary based qualification in health professional education or similar. Research on clinical supervision in podiatry practice is in a very early phase of development globally. While the present findings validate our results of prior research and provide additional details about the nature of clinical supervision, further investigations are needed to enhance our understanding of this critical activity and to better prepare our new graduates, albeit safely, competently and confidently.

## Supplementary Information

**Additional file 1:** **Appendix 1.** Survey instrument.

## Data Availability

Authors can confirm that all relevant data are included in the article and/or its supplementary information files.
