# Supplementary material for: Clinical supervision in podiatry in Australia and New Zealand: supervisor challenges in this role
Source: BMC Med Educ. 2023 Feb 9;23:104. doi: 10.1186/s12909-023-04056-z (PMC9910774; doi:10.1186/s12909-023-04056-z)
Supplement: Supplementary file 1 — Additional file 1: Appendix 1. Survey instrument. [file 12909_2023_4056_MOESM1_ESM.docx]

**Appendix 1. Survey instrument**

**Clinical supervision**

**Demographics**

- Gender, age group, year of graduation, initial podiatry qualifications and location, other formal qualifications, postcode of practice.

**Section 1 – Status as a clinical supervisor**

- Are you or have you been a clinical supervisor of podiatry students on placement? (Q20)
- Yes, No
- Are you interested in becoming a clinical supervisor of podiatry students? (Q21)
- Yes, No, Maybe
  - Explain your choice

**Section 2 – Experience as a clinical supervisor**

- How long have you been a clinical supervisor of podiatry students? (Q22)
- Less than one month, 1-6 months, 6-12 months, 1-2 years, 2-5 years, more than 5 years
- When was the last time you provided podiatry student clinical supervision? (Q23)
- In the past week, in the past month, 2-3 months ago, 4-6 months ago, 7-12 months ago, 1 or more years ago
- Do you plan to continue supervising podiatry students? (Q24)
- Yes, No, Unsure
  - Explain your choice
- In what work setting(s) do/did you primarily supervise podiatry students? (Q25)
- Public sector hospital, private sector hospital, private practice, community health centre, university student clinic
- Is the clinical supervision of students expected of you in your workplace(s)? (Q26)
- Yes, No
  - Other comments
- What year level of student(s) do/did you predominantly supervise? (Q27)
- 1^st^ year, 2^nd^ year, 3^rd^ year, 4^th^ year, unsure
  - Please explain
- In what degree qualification(s) are/were the podiatry students principally enrolled? (Q28)
- Bachelor, Honours, Graduate-entry (Bachelor), Graduate-entry Masters, Masters, Doctoral (Doctor of Podiatric Medicine), Unsure
  - Please explain
- When you commenced supervision, how prepared were you to supervise podiatry students? (Q29)
- Not at all prepared, Somewhat unprepared, Somewhat prepared, Very prepared
  - Explain your choice
- Did you receive any training or educational support to assist you in your role as a clinical supervisor? (Q30)
- Yes, No
  - Explain your choice
- In your opinion, whose responsibility is it to ensure quality clinical supervision of podiatry students? (Q31)
- Broader podiatry profession, placement site, student, supervisor, university
- If you have supervised students in both the public and private sector, in your opinion, which is the better environment to supervise students? (Q32)
- Public, private, there is no difference, unsure, not applicable

**Section 3 – Previously identified supervisory challenges (Q33 – 38)**

- Strongly disagree, Disagree, Neither agree nor disagree, Agree, Strongly Agree
- Please rate your perception of the following challenges that have been identified by clinical supervisors (Q33, 5 items):

*Supervisor-specific:*

1. Supervision of students is time-consuming
2. When providing patient care, it is difficult to balance being a good clinician and an effective supervisor
3. Supervisors experience fatigue, stress and burnout from students
4. Patients become fatigued from students
5. It is difficult to keep up-to-date with own knowledge and skills in clinical practice to be an effective supervisor

- Please rate your perception of the following student-related issues identified by clinical supervisors (Q34, 3 items):

*Students:*

1. Lack interest and motivation on placement (e.g. marking time)
2. Have unrealistic expectations about their intended learning during placement
3. Display a “know it all already” attitude on placement

- Please rate your perception of the following issues specific to guidelines and processes for supervision (Q35, 7 items):

*Supervisor-specific:*

1. Universities do not provide clarity of the supervisory role, related responsibilities and processes
2. Lack of standardised assessment criteria between universities creates difficulties for supervisors
3. Lack of formal and/or informal training contributes to uncertainty as a supervisor
4. There is limited workplace support and/or resource for supervisory role
5. There is limited university support and/or resource for supervisory role

*Curriculum:*

1. Placement is too short (e.g. 1-2 weeks)
2. Placement is too long (e.g. 4-6 weeks)

- Please rate your perceptions of the following identified deficiencies specific to self-efficacy and organisational skills of students on placement (Q36, 5 items):

*Students:*

1. Lack confidence in their clinical skills
2. Display difficulty ‘fitting in’ the clinical setting with staff and patients
3. Have poor time management skills
4. Have limited ability to prioritise patient care
5. Lack independence when providing patient care

- Please rate your perceptions of the following identified deficiencies related to clinical and communication skill development of students on placement (Q37, 5 items):

*Students:*

1. Are unable to communicate effectively with supervisors, other staff and patients
2. Have difficulty establishing rapport with patients
3. Show limited ability to carry out basic manual podiatry clinical skill procedures

*Curriculum:*

1. Students have limited ‘hands-on’ patient contact in private practice placements which impacts student learning
2. Students are being exposed to more public sector “hands-on” placements which is causing an imbalance in learning

- Please rate your perception of the following identified curriculum issues (Q38, 4 items)

1. There is poor alignment of placement for the student’s stage of learning
2. Clinical placement occurs too late in the curriculum
3. Students have limited contact with patients (despite the prescribed 1000 hours of placement)
4. There is a lack of training in small business administration skills for practice
